# ERV1 Overexpression in Myeloid Cells Protects against High Fat Diet Induced Obesity and Glucose Intolerance

**DOI:** 10.1038/s41598-017-13185-7

**Published:** 2017-10-09

**Authors:** Corneliu Sima, Eduardo Montero, Daniel Nguyen, Marcelo Freire, Paul Norris, Charles N. Serhan, Thomas E. Van Dyke

**Affiliations:** 1 Center for Clinical and Translational Research, The Forsyth Institute, 245 First Street, Cambridge, MA 02138 USA; 2 Department of Oral Medicine, Infection and Immunity, Harvard School of Dental Medicine, 188 Longwood Ave, Boston, MA 02115 USA; 3 Section of Graduate Periodontology, Faculty of Odontology, University Complutense of Madrid, Pza. Ramón y Cajal s/n, Madrid, 28040 Spain; 4 Center for Experimental Therapeutics and Reperfusion Injury, Department of Anesthesiology, Perioperative and Pain Medicine, Brigham and Women’s Hospital, and Harvard Medical School, 60 Fenwood Road, Boston, MA 02115 USA

## Abstract

Non-resolving inflammation is a central pathologic component of obesity, insulin resistance, type 2 diabetes and associated morbidities. The resultant hyperglycemia is deleterious to the normal function of many organs and its control significantly improves survival and quality of life for patients with diabetes. Macrophages play critical roles in both onset and progression of obesity-associated insulin resistance. Here we show that systemic activation of inflammation resolution prevents from morbid obesity and hyperglycemia under dietary overload conditions. In gain-of-function studies using mice overexpressing the human resolvin E1 receptor (ERV1) in myeloid cells, monocyte phenotypic shifts to increased patrolling-to-inflammatory ratio controlled inflammation, reduced body weight gain and protected from hyperglycemia on high-fat diet. Administration of a natural ERV1 agonist, resolvin E1, recapitulated the pro-resolving actions gained by ERV1 overexpression. This protective metabolic impact is in part explained by systemic activation of resolution programs leading to increased synthesis of specialized pro-resolving mediators.

## Introduction

Temporal and spatial imbalance in recruitment of monocytes/macrophages and their phenotypic polarization across the M1 (pro-inflammatory) - M2 (pro-resolution) spectrum has been implicated in inflammation resolution failure and chronicity in numerous systemic conditions including obesity associated insulin resistance and type 2 diabetes^1–4^. Polarization of adipose tissue macrophages (ATM) to M1 plays a critical role in onset of insulin resistance^5^. A switch from a protective M2 to M1 phenotype in ATM is central to inflammation-mediated insulin resistance. Visceral adipose tissue (VAT) is the main driver of metabolic syndrome progression^6,7^. VAT expansion is associated with local production of monocyte chemoattractant protein 1 (MCP-1, also known as chemokine ligand 2 or CCL2) that facilitates infiltration of inflammatory monocytes via chemokine receptor 2 (CCR2)^8,9^. Similarly, obesity induces hepatic recruitment of monocytes via CCR2 promoting steatosis and insulin resistance^10,11^. The relative contribution of monocyte activation to an M1 phenotype and switching of polarization from M2 to M1 induced by local tissue signals in obesity is inconclusive. The origins and dynamics of monocytes and tissue macrophages in steady state compared to pro-inflammatory conditions are likely different^12,13^. Understanding the mechanisms governing monocyte dynamics in regulation of immunity and restoration of homeostasis is critical to develop monocyte-targeted therapeutics for management of obesity associated inflammation and metabolic imbalance.

In humans and mice, two different monocyte populations, with distinct surface chemokine receptor profiles – CCR2^+^CX_3_CR1^int^ (inflammatory) and CCR2^-^CX_3_CR1^high^ (patrolling), share M1-like and M2-like phenotypes, respectively, which suggests that M1 and M2 use different modes of tissue trafficking in inflammation and resolution^14–16^. While CCR2^+^CX_3_CR1^int^ monocytes reach inflammatory sites and give rise to conventional dendritic cells and M1 macrophages, CCR2^-^CX_3_CR1^high^ monocytes infiltrate tissues and differentiate into M2 macrophages^17^. It has been suggested that enhanced recruitment of CCR2^-^CX_3_CR1^high^ monocyte and M2 polarization in VAT may contribute to resolution of inflammation and prevention of glucose intolerance in the context of dietary overload^4,18^. Mounting evidence suggests that peroxisome proliferator-activated receptor gamma (PPAR-γ) controls the inflammatory potential of monocytes/macrophages, with net impact on the course of obesity-associated inflammation^19–21^. These actions are attributed in part to CX_3_CR1 upregulation and CCR2 downregulation by PPAR-γ activation, though it is not clear whether CX_3_CR1 and CCR2 recruit only M2 and M1 macrophages, respectively^19^. Deletion or inhibition of PPAR-γ leads to impaired maturation of M2 macrophages, exacerbation of diet-induced obesity, VAT inflammation, insulin resistance, and glucose intolerance^21,22^. Further, PPAR-γ agonists promote redistribution of lipids toward adipocytes and extend the M2 ATM polarization state, preventing the lipid alterations associated with M1 ATM polarization^23^. Importantly, PPAR-γ-dependent polarization of M2 macrophages occurs at the level of the monocyte as demonstrated by upregulation of M2 markers by peripheral blood monocytes and the reduction in systemic M1-derived soluble factors after treatment with thiazolidinediones^24,25^. Altogether these findings suggest that induction of pre-M2 macrophage phenotype in monocytes has the potential to regulate non-resolved inflammation associated with obesity thus preventing insulin resistance and associated morbidities.

One approach to gain pro-resolution function in monocytes/macrophages is to stimulate systemic M2 polarization with therapeutic delivery of ω-3 polyunsaturated fatty acids (PUFAs) or their active metabolites, the specialized pro-resolving mediators (SPMs) including resolvins, protectins and maresins^26^. ω-3 PUFAs and derived SPMs are agonists of G-protein coupled receptors (GPCRs) GPR32/DRV1 (Resolvin D1 Receptor), GPR18/DRV2 (Resolvin D2 Receptor), GPR120 (ω-3 PUFAs receptor), and ChemR23/ERV1 (Resolvin E1 Receptor)^26–29^. Bioactive mediators derived from ω-3 PUFAs, SPMs, including the resolvins E1 (RvE1) and D1 (RvD1), protectin D1 (PD1) and maresin 1 (MaR1) were found to limit obesity associated inflammation, switch macrophage polarization to M2 and improve insulin sensitivity in type 2 diabetes^26,30–33^. Knockout of ERV1 in mice was associated with reduced adiposity and impaired glucose tolerance suggesting that this receptor plays an important role in adipose tissue development, inflammation and glucose homeostasis^34^. Recent studies also demonstrated that GPR120 mediates potent anti-inflammatory, pro-resolution and insulin sensitizing actions *in vivo*^35,36^. However, the limited rate of gut absorption and incorporation into cell membranes in the context of relatively high doses necessary to achieve biological benefits makes pharmacological administration of ω-3 PUFAs challenging^37,38^. Although synthesis of eicosapentaenoic (EPA) and docosahexaenoic acids (DHA) in humans is possible by conversion of dietary alpha linolenic acid (ALA), the conversion rate is below 20% and significantly lower in males compared to females^39^. As opposed to their precursors DHA and EPA, SPMs synthesized by leukocytes and endothelial/epithelial cells from dietary PUFAs exert their biological actions at the picomolar and nanomolar range through autocrine and paracrine actions via specific GPCRs^28,40^. Thus, activation of resolution programming through enhanced expression of SPM receptors by myeloid cells provides a rationale for gain-of-function studies to limit inflammation and improve insulin sensitivity in diet induced obesity and insulin resistance.

To determine the impact of monocyte/macrophage ERV1 overexpression on insulin resistance in obesity, mice conditionally overexpressing the human ERV1 receptor in myeloid cells (ERV1 transgenic or ERV1tg) were compared to wild type mice in a high-fat diet over-nutrition model^41^. Here we report that ERV1tg mice on high fat diet (HFD) for 20 weeks are protected from morbid body weight gain, fasting hyperglycemia and inflammation in VAT and liver. ERV1tg mice have increased numbers of patrolling peripheral blood monocytes, decreased CCR2 expression on monocytes and produce higher amounts of SPMs, both in steady-state and under dietary overload when compared to wild type mice.

## Methods

### Mice and Diet

Wild type FVB mice were purchased from The Jackson Laboratory (Bar Harbor, ME). We previously generated conditional ERV1 (aka ChemR23 or CMKLR1) transgenic (ERV1tg) mice on the FVB background using a construct linked to CD11b promoter to limit expression to myeloid cells^42,43^. The CD11b promoter directs high-level macrophage expression of reporter genes in transgenic mice^44^. All mouse experiments were in conformity with the standards of the Public Health Service Policy on Human Care and Use of Laboratory Animals and were approved by the Institutional Animal Care and Use Committee of The Forsyth Institute. Mice were maintained in a temperature-controlled room (22 ± 1 °C) with a 12 h light/dark cycle and ad libitum access to food and water. Six-week-old mice on a standard chow diet (NCD; 13% kcal from fat, 25% kcal from protein, 62% kcal from carbohydrate; LabDiet, St. Louis, MO, USA), were randomly allocated to remain on the NCD or to receive a high fat diet (HFD; 60% kcal from fat, 20% kcal from protein, 20% kcal from carbohydrate; D12492, ResearchDiets, New Brunswick, NJ, USA) ad libitum for 20 weeks.

### Metabolic Studies and Histology

Mice were fasted between 10 a.m. and 5 p.m. for fasting glucose (FG), insulin (FI), glucose tolerance tests (GTTs) and insulin tolerance tests (ITTs). GTTs and ITTs were performed as previously described^41^. Fasted mice were administered D-glucose (1 g/kg body weight) or human insulin (Humulin R, Eli Lilly, Cambridge, MA, USA) (1 U/kg body weight) intraperitoneally and blood glucose levels measured at indicated time points (Accu-Chek Nano, Roche Diagnostics, Indianapolis, IN, USA). Fasting serum insulin was measured by ELISA (CrystalChem, Downers Grove, IL, USA). The quantitative insulin-sensitivity check index (QUICKI) was calculated as 1/[log(FG) + log(FI)]. 5 µm sections of paraffin embedded visceral adipose tissue (VAT) and liver samples were stained with hematoxylin and eosin. Photomicrographs of stained slides were captured with a Zeiss Cell Observer Z widefield using ZEN v2.3 software (Carl Zeiss Microscopy, Thornwood, NY, USA). Fat droplet size in liver and adipocyte size in VAT were measured using the Adiposoft plugin for Fiji (NIH).

### RNA Extraction and Real-Time qPCR

Total RNA was extracted from liver and epididymal fat pads using TRIzol reagent (Invitrogen, Carlsbad, CA, USA) per the manufacturer’s protocol, and reverse transcribed using a high-capacity cDNA reverse transcription kit with RNase inhibitor (Applied Biosystems, Foster City, CA, USA). Samples were assayed on a GeneAmp 9700 PCR thermocylcer at 25 °C for 10 min, 37 °C for 120 min, and 85 °C for 5 min (Applied Biosystems). Real-time qPCR was performed using TaqMan fast advanced master mix (Applied Biosystems) and pre-designed target and endogenous TaqMan gene primer/probe assays (Thermo Fisher Scientific, Waltham, MA, USA) to determine the amounts of inflammatory (*NLRP3*, *TNF-α*, *IL-6*, *IL-10*, *IL-1RA*), glucose (*GLUT-2*, *GLUT-4*) and insulin (*INSR*, *IRS-1*) response, and lipid metabolism (*CEBP-α*, *SREBP1*, *SREBP2*, *LXR-α*, *LXR-β*, *FABP4*, *PPAR-α*, *PPAR-γ*) mRNA (Table S1). Four different endogenous control genes (*GAPDH*, *18S*, *β-actin* and *HPRT*) were screened on all samples to provide an optimal normalized dataset. Each sample was run in duplicate, and the level of gene expression was normalized to the housekeeping gene *β-actin*. Of all endogenous control genes expression of *β-actin* did not differ significantly between WT and ERV1tg mice (P > 0.20). The reaction was carried out in a StepOnePlus real-time PCR system (Applied Biosystems) set at 50 °C for 2 min, 95 °C for 20 seconds, then 40 cycles of 95° for 1 sec, 60° for 20 sec. CT values were calculated from the StepOnePlus Software v2.3 (Applied Biosystems). Changes in gene expression were calculated by the 2(−∆∆C(T)) method using mean cycle threshold (CT) values. Fold changes in gene expression were assessed by calculating the difference between the CT values of *β-actin* mRNA and that of the target gene (∆CT). The mean ∆CT of healthy WT were subtracted from individual ∆CT values to obtain ∆∆CT. Fold changes in gene expression were calculated using the equation 2^−∆∆CT^ for each experimental condition using healthy WT as reference.

### Immunofluorescence and Immunohistochemistry

Double immunofluorescence antigen labeling of the paraffin embedded mouse visceral adipose tissue (VAT) and pancreas was performed with antibodies against F4/80, CD206, iNOS (for VAT), glucagon or insulin (for pancreas) following antigen retrieval (10 mM sodium citrate pH6, 1 mg/ml sodium borohydride; ICN chemicals) and blocking of nonspecific binding (5% normal donkey serum, Jackson ImmunoResearch Lab Inc, West Grove PA, USA). Slides were then incubated with rat anti-F4/80 (1:200, BioRad) and rabbit anti-CD206 (1:500, Abcam, Cambridge, MA, USA) or rat anti-F4/80 (1:200, BioRad) and rabbit anti-iNOS (1:100, BD) or rabbit anti-Glucagon (1:400, Cell Signaling Technology) and Guinea pig anti-Insulin (1:500, Abcam) or rabbit anti-TNF-α (1:100, Abcam) overnight at 4 °C. The slides were washed three times with TBS and incubated with CY3 conjugated Donkey anti-rabbit and Alexa 647 conjugated donkey anti-rat secondary antibodies (Jackson ImmunoResearch Lab, 1:300). Samples were then washed three times with TBS and the slides were mounted with Prolong Gold anti-fade mounting media containing DAPI (Invitrogen). Photomicrographs of stained slides were captured with a Zeiss Zeiss LSM 780 confocal microscope using ZEN v2.3 software (Carl Zeiss Microscopy). For immunohistochemistry for ERV1, YM1 and CD163 expression in liver and VAT antigen retrieval (10 mM Na citrate pH 6.0 in microwave) of de-paraffinized sections was followed by blocking of nonspecific binding (1.5% normal goat serum) (Jackson ImmunoResearch Lab Inc). Slides were then incubated overnight with primary rabbit anti-mouse YM1 (1:500) or CD163 (1:500) (Abcam, Cambridge, MA, USA) or mouse anti-human ChemR23 (ERV1) (1:3000) (R&D Systems, Minneapolis, MN, USA) antibodies, followed by incubation with biotinylated secondary anti-rabbit antibody and coverslipped with permount (Thermo Fisher Scientific).

### Flow Cytometry

Whole peripheral blood (40 µl) collected by tail venipuncture was analyzed for myeloid cell numbers and immunophenotyping by acoustic focusing flow cytometry (Attune NxT, Thermo Fisher Scientific). Uncoagulated blood (4% EDTA) was blocked with 1% bovine serum albumin and incubated with PE conjugated anti-CD11b (1:100), eFluor450 conjugated anti-Ly6C (1:100), eFluor780 conjugated anti-CD115 (1:100) (eBioscience, San Diego, CA, USA), APC conjugated anti-CCR2 (1:100) or APC conjugated anti-CX_3_CR1 (1:100) or APC conjugated anti-ERV1 (1:100)(R&D Systems, Minneapolis, MN, USA) antibodies for 1 hour and diluted 1:400 for partial red blood cell (RBC) lysis (High-Yield Lyse 1:2 in phosphate buffer saline) (Invitrogen). Calibration was performed using antibodies conjugated to magnetic beads (OneComp eBeads, eBioscience). Leukocytes were selected on side scatter (SSC) Blue vs. SSC-Violet by excluding remaining RBCs. Doublets were excluded on forward scatter (FSC) height (H) vs. FSC width (W) dot plots (<20%). Each sample was analyzed in duplicate. Data were analyzed using the Attune NxT Software v2.4 and FlowJo v10.

### Lipid Mediator Metabololipidomics

Serum and adipose tissues were snap-frozen in liquid nitrogen and stored at −80 °C prior to solid-phase extraction (SPE) of lipid mediators and LC-MS/MS metabololipidomics. Internal standards d8–5-HETE, d5-RvD2, d5-LXA_4_, d4-LTB_4_, d4-PGE_2_ (500 pg each; Cayman Chemical, Ann Arbor, MI) were added along with four volumes of methanol to facilitate protein precipitation; adipose tissue was homogenized using a glass dounce. After centrifugation at 1,000 × g, 4 °C for 5 min, samples were then brought down to ≤10% methanol and loaded onto solid-phase extraction (SPE) Isolute C18 SPE 3 mL, 100 mg cartridges (Biotage, Charlotte, NC USA) following rapid acidification (<30 seconds) to ~pH 3.5. Before elution, lipid mediators bound to SPE matrix were neutralized with double distilled H_2_O. Methyl formate fractions from SPE were brought to dryness under a gentle stream of nitrogen and resuspended in 1:1 methanol:water before injection into a liquid chromatography-tandem mass spectrometry system consisting of a QTrap 5500 (AB Sciex) equipped with a Shimadzu LC-20AD HPLC (Tokyo, Japan). A Poroshell 120 EC-18 column (100 mm × 4.6 mm × 2.7 μm; Agilent Technologies, Santa Clara, CA, USA) was kept in a column oven maintained at 50 °C, and lipid mediators (LMs) were eluted in a gradient of methanol/water/acetic acid from 55:45:0.01 (v/v/v) to 98:2:0.01 at 0.5 mL/min flow rate. In order to monitor and quantify the levels of targeted LMs, multiple reaction monitoring (MRM) was used with MS/MS matching signature ion fragments for each molecule (at least six diagnostic ions; ~0.1 pg limits of detection)^45^.

### Statistics

Data are mean values ± SEM of the indicated number of experiments. Significance was tested using either one way ANOVA for repeated measurements or unpaired two-tailed *t* tests and Holm-Sidak correction for multiple comparisons assuming unequal variance per time point or experimental condition, with α = 0.05 applied, as indicated. All statistical analyses were performed using GraphPad Prism 7 (GraphPad Software, La Jolla, CA, USA).

## Results

### Myeloid ERV1 protects from diet induced obesity, hepatic steatosis and glucose intolerance

**Figure 1:**
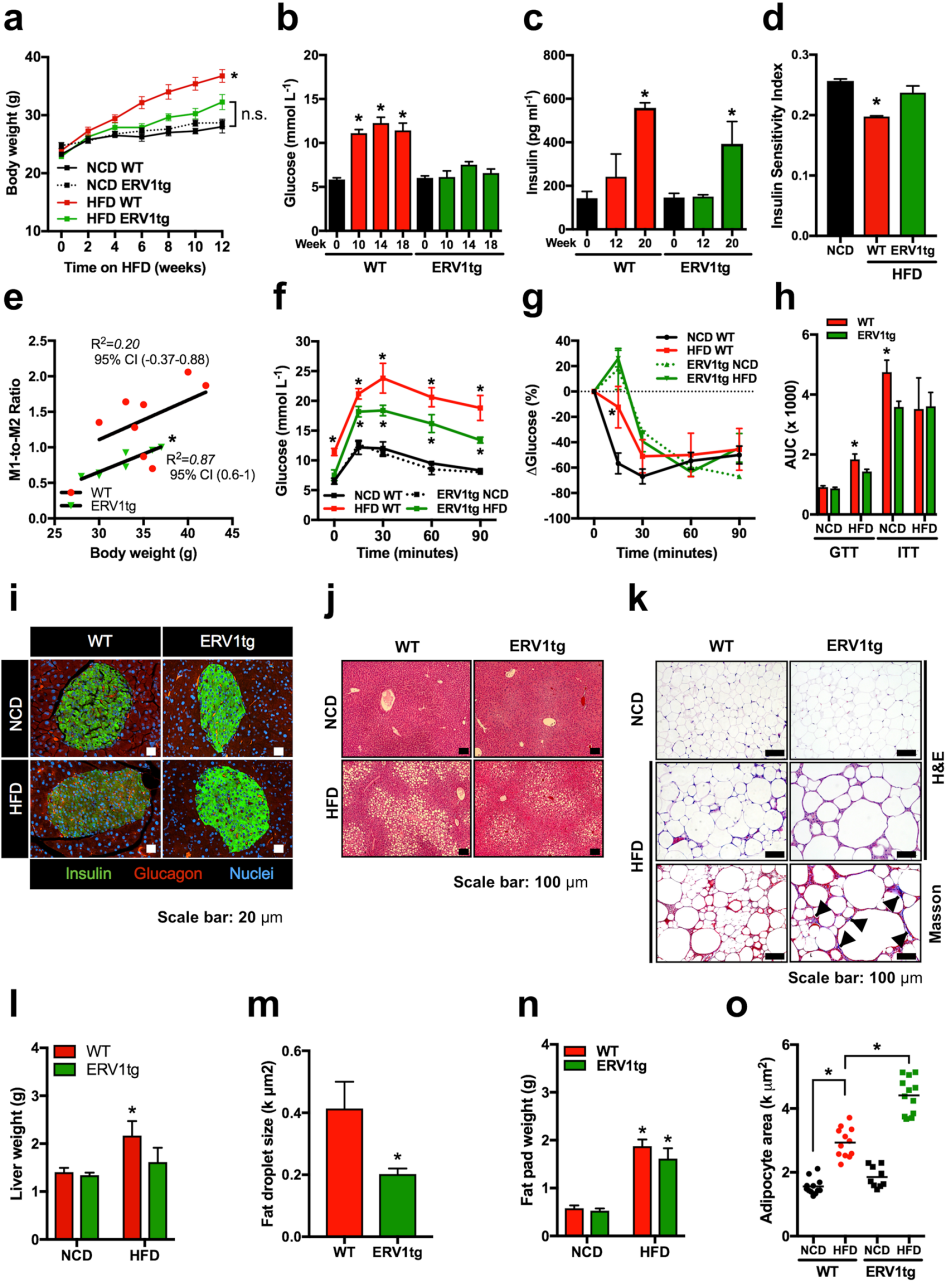
ERV1 transgenic mice are protected from diet induced obesity, hepatic steatosis and glucose intolerance. (**a**) Body weights of ≥6 weeks-old male FVB mice on normal control diet (NCD) or high fat diet (HFD) for 12 weeks (n = 12 NCD per group; *p = 0.0075, ANOVA repeated measures test; F (1.126, 6.755) = 13.56). (**b**,**c**) Fasting blood glucose and serum insulin were measured in WT (n = 12) and ERV1tg (n = 8) mice for 20 weeks on HFD (*p < 0.05, unpaired *t-*tests vs. baseline). (**d**) Quantitative insulin sensitivity check index (QUICKI) for mice on NCD or HFD for 16 weeks (n = 8 mice/group, *P < 0.05, *t* test). (**e**) Correlation between fasting blood glucose and body weight in mice on NCD or HFD for 16–20 weeks (*Pearson, *P* < 0.01, n = 12 mice/group). (**f**,**g**) Glucose (**f**) and insulin (**g**) tolerance in NCD- or HFD-fed WT and ERV1tg mice (n = 4 per group, *P < 0.05 HFD vs. NCD, *t* tests, Holm-Sidak correction for multiple comparisons assuming unequal variance per time point (*P* < 0.01), one of two similar experiments). (**h**) AUC for glucose (GTT) and insulin (ITT) tolerance test results for WT and ERV1tg mice (n = 4 per group, *P < 0.05 HFD vs NCD, *t* tests). (**i**) Representative glucagon and insulin immunofluorescence micrographs of pancreatic islets from WT and ERV1tg mice on NCD or HFD for 18 weeks. (**j**,**k**) Representative micrographs of liver (**j**) and VAT (**k**) stained with hematoxylin and eosin, and Masson’s trichrome (**k**, *Bottom row*; arrows indicate collagen). (**l**,**n**) Weights of livers (**l**) and single epididymal VAT (**n**) of WT and ERV1tg mice on NCD or HFD for 18 weeks (n = 4 mice/group, *P < 0.05 HFD vs. NCD, *t* test). (**m**) Fat droplet size in liver of WT and ERV1tg mice on HFD for 18 weeks (n = 4 mice per group, *P < 0.05, *t* test). (**o**) Adipocyte size in VAT of mice on HFD or NCD (n = 4 per mice group, *P < 0.05 *t* tests). All *t* tests were unpaired and two tailed. Histograms represent mean ± SEM.

To test whether overexpression of human ERV1 by myeloid cells results in gain of pro-resolution function in these cells in the context of diet-induced obesity, ERV1tg and WT mice were fed HFD or NCD from 6 weeks of age for 20 weeks. Despite similar food intake male ERV1tg mice gained significantly less weight, did not develop fasting hyperglycemia and had normal insulin sensitivity after 5 months of HFD feeding compared to WT mice (Fig. 1a–d). Fasting glucose levels correlated strongly with body weight in WT mice but not ERV1tg suggesting physiologic fat accumulation in the latter (Fig. 1e). Female mice did not gain significant weight or develop hyperglycemia after 8 months on HFD (Figure S1). Despite normal fasting glucose levels male ERV1tg developed some glucose intolerance upon intraperitoneal glucose administration compared to chow normal control diet (NCD) and delayed response to insulin. Interestingly, the delayed response to insulin was also observed in ERV1tg on NCD suggesting either different regulation of glucose and lipid metabolism or insulin absorption compared to WT mice (Fig. 1f–h).

To get further insight into these metabolic differences we assessed the pancreas for insulin and glucagon producing islet cells, and liver and epididymal visceral adipose tissues (VAT) for lipid accumulation. Numbers of insulin positive cells were decreased and those of glucagon positive cells increased in islets of WT on HFD whereas ERV1tg maintained a high proportion of insulin positive cells under dietary overload (Fig. 1i). The islet size was also reduced in WT but not ERV1tg mice (Figure S4a,b). This is consistent with previous findings that chronic hyperglycemia leads to a dramatic change in insulin to glucagon ratio and β-cell apoptosis in pancreatic islets^46,47^. Our results indicate that ERV1tg may respond promptly to transient hyperglycemia through increased insulin secretion. Fasting glucose levels are unaltered when on HFD for months and fasting insulin levels are increased after 20 weeks of HFD feeding (Fig. 1b,c). ERV1tg mice on HFD had similar VAT expansion but no significant increase in liver size or hepatic steatosis compared to WT, indicating that accumulation of fat in liver was a major contributor to the weight gain difference between genotypes (Fig. 1k–n). Despite similar VAT size adipocytes of ERV1tg on HFD were significantly larger compared to WT suggestive of predominantly hypertrophy over hyperplasia. Additionally, peri-adipocyte fibrosis was evident in ERV1tg but not WT (Fig. 1k,o). Hepatic glycogen depletion during fasting was reduced in ERV1tg mice on either diet compared to WT (Figure S4c).

**Figure 2:**
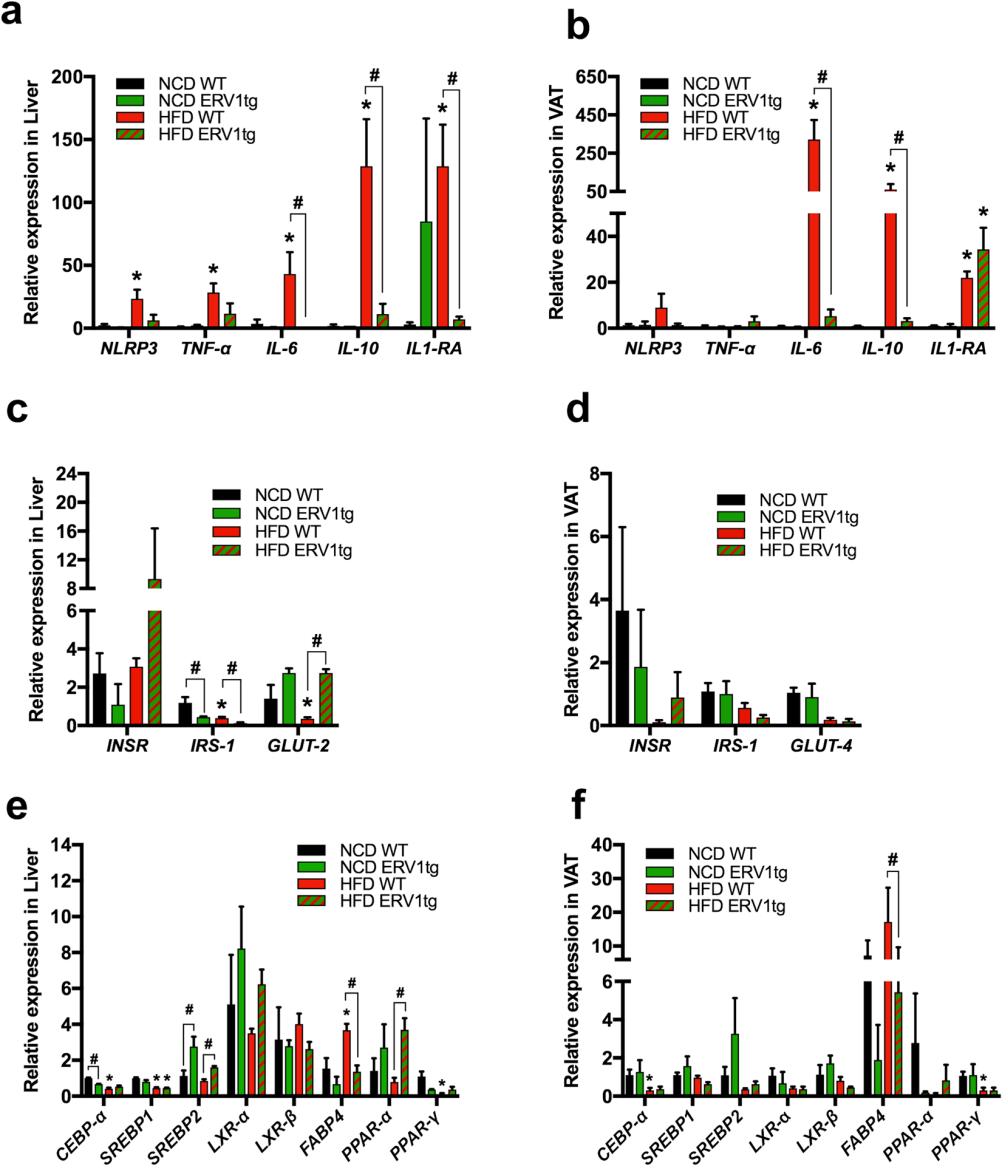
Myeloid ERV1 overexpression favors protective expression of inflammatory, glucose and lipid metabolism genes in liver and adipose tissue under dietary fat overload. To interrogate the impact of myeloid ERV1 overexpression during dietary fat overload on expression of inflammatory, adiposity and glucose response gene in central metabolic organs, liver and VAT samples from ERV1tg and WT male mice on HFD or NCD for 20 weeks was assessed by RT-qPCR. Expression of inflammatory genes (*NLRP3*, *TNF-α*, *IL-6*, *IL-10* and *IL-1RA*), glucose (*GLUT-2*, *GLUT-4*) and insulin (*INSR*, *IRS-1*) response genes, and lipid metabolism genes (*CEBP-α*, *SREBP-1*, *SREBP-2*, *LXR-α*, *LXR-β*, *FABP4*, *PPAR-α* and *PPAR-γ*) in liver (**a,c,e**) and VAT (**b,d,f**) was measured relative to β-Actin. Expression was normalized to WT NCD (n = 4 mice per group, *P < 0.05, HFD vs. NCD, ^#^*P* < 0.05, ERV1tg vs. WT, unpaired two tailed *t* tests). Samples were run in duplicate. Histograms represent mean ± SEM. GLUT, glucose transporter; INSR, insulin receptor; IRS, insulin receptor substrate; CEBP-*α*, CCAAT/enhancer-binding protein alpha; SREBP, sterol regulatory element binding proteins; LXR, liver X receptor; FABP, fatty-acid-binding proteins; PPAR, peroxisome proliferator-activated receptors.

To validate these changes in key metabolic organs and assess whether reduced inflammation underlies the protective role of ERV1 overexpression in myeloid cells, we performed quantitative PCR on liver and VAT tissues from mice on HFD for 20 weeks. Indeed, the expression of pro-inflammatory genes (*NLRP3*, *TNF-α*, *IL-6*) in liver and VAT of HFD fed ERV1tg mice was not significantly increased, while that of the anti-inflammatory gene *IL1-RA* was increased in VAT of HFD fed mice of both genotypes compared to NCD. *IL-6* and *IL-10* was downregulated in ERV1tg mice on HFD compared to WT (Fig. 2a,b). The expression of *IRS-1* was downregulated and that of *GLUT-2* upregulated in liver of ERV1tg on HFD compared to WT (Fig. 2c) and no significant differences were noted in VAT between ERV1tg and WT (Fig. 2d). In WT mice on HFD, *CEBP-α* and *PPAR-γ* were downregulated in both liver and VAT, and *FABP4* was upregulated in liver compared to NCD. In liver of ERV1tg mice *SREBP2* was upregulated both on NCD and HFD when compared to WT, and *PPAR-α* was upregulated on HFD when compared to WT. In VAT of ERV1tg mice *FABP4* was downregulated on HFD when compared to WT (Fig. 2e,f). Taken together these findings indicate that myeloid ERV1 overexpression prevents induction of inflammatory genes in central metabolic organs and associated overt inflammation, and downregulates genes involved in liver fat deposition under dietary fat overload.

### The increased M2-to-M1 monocyte ratio in ERV1tg mice is maintained on HFD and strongly correlates with body weight

**Figure 3:**
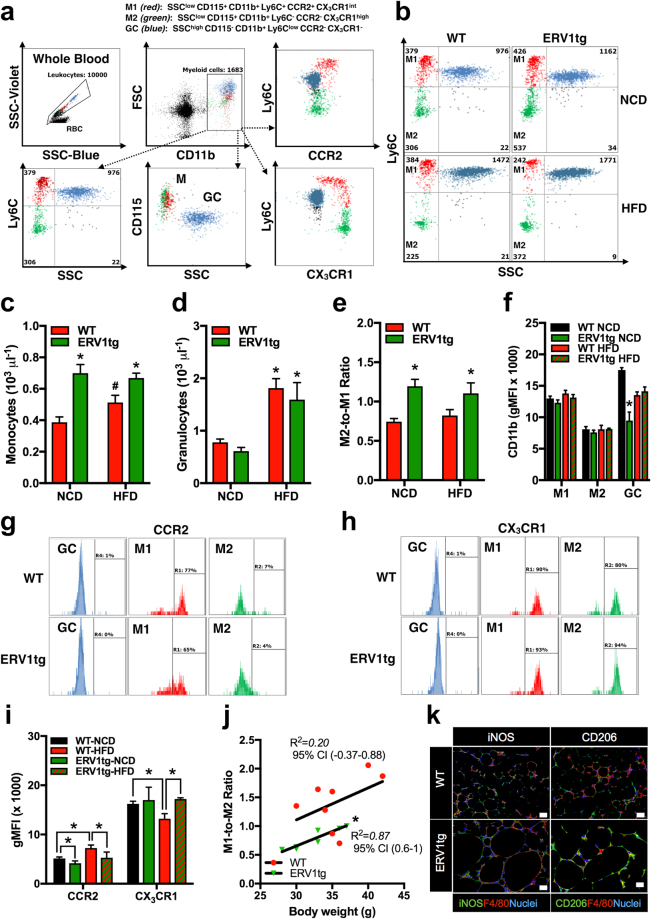
ERV1tg mice maintain peripheral blood monocyte and VAT macrophage skewing to pro-resolution (M2) phenotype under dietary overload conditions. Peripheral blood myeloid cell immunophenotyping was performed for age-matched male mice on NCD or HFD for 16 weeks (n = 8 per group for NCD, n = 4–8 per group for HFD) (**a**) Gating strategy for peripheral blood monocyte immunophenotyping by acoustic focusing flow cytometry. Monocytes were identified as SSC^low^CD115^+^CD11b^+^ and granulocytes (GC) as SSC^high^CD115^−^CD11b^+^. Monocytes were further identified as M1-like (Ly6C^+^CCR2^+^CX_3_CR1^int^) or M2-like (Ly6C^−^CCR2^−^CX_3_CR1^high^) (**b**) Representative dot plots of granulocytes, M1- and M2-like monocytes. Numbers represent absolute counts per 10000 leukocytes. (**c**) Monocyte counts (*P < 0.05, *t* tests ERV1tg vs WT; ^#^*P* < 0.05, *t* tests HFD vs NCD). (**d**) Granulocyte counts (*P < 0.05, *t* tests HFD vs NCD). (**e**) M2-to-M1 monocyte ratio (*P < 0.05, *t* tests ERV1tg vs WT). (**f**) CD11b expression (*P < 0.05, one-way ANOVA). (**g**,**h**) Representative histograms of CCR2 (**g**) and CX_3_CR1 (**h**) surface expression in peripheral blood GC, M1 and M2 monocytes. Numbers represent percentage of cells with geometric mean fluorescence intensity (gMFI) > 1000. (**i**) CCR2 and CX_3_CR1 receptor expression on peripheral blood monocytes (SSC^low^CD115^+^CD11b^+^) (*P < 0.05, *t* tests). (**j**) Correlation between monocyte M1-to-M2 ratio and body weight in mice on NCD or HFD (*Pearson, *P* < 0.01, n = 8 mice per group). (k) Representative immunofluorescence micrographs of M1 (iNOS) and M2 (CD206) macrophages in VAT of WT and ERV1tg mice on NCD or HFD for 20 weeks. All *t* tests were unpaired and two tailed. Histograms represent mean ± SEM.

**Figure 4:**
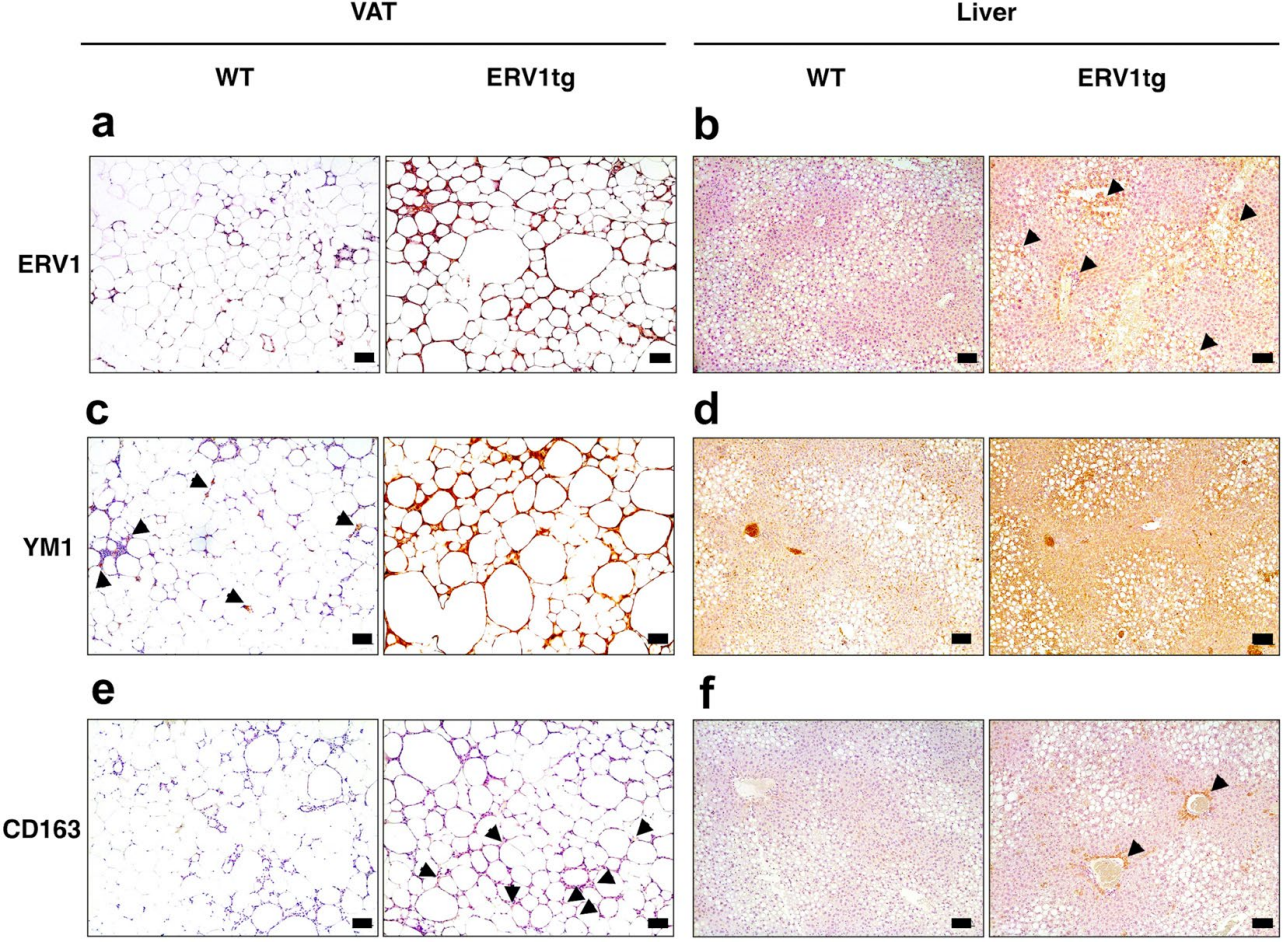
ERV1 and M2 markers YM1 and CD163 are increased in metabolic tissues of ERV1tg male mice. VAT and liver samples from WT and ERV1tg male mice fed a HFD or NCD for 20 weeks were assessed by immunohistochemistry for ERV1, YM1 and CD163 expression. Representative micrographs of VAT (**a**) and liver (**b**) stained for ERV1. High ERV1 expression was noted in crown like structures throughout the VAT, around fat droplets and surrounding portal vein areas (*arrows*) of ERV1tg mice. Representative micrographs of VAT (**c**) and liver (**d**) stained for M2 marker YM1. Similar to ERV1, YM1 was highly expressed in both VAT and liver of ERV1tg mice compared to WT (*arrows* indicate YM1 positive cells). Representative micrographs of VAT (**e**) and liver (**f**) stained for M2 marker CD163. Higher expression was noted in ERV1tg mice in VAT (arrows) and liver, particularly in areas surrounding portal veins (*arrows*). Scale bar, 100 μm.

Since inflammatory monocytes recruit via CCR2 in response to CCL2 (MCP-1) in adipose tissue during pathogenic expansion leading to insulin resistance, we next determined whether peripheral blood monocytes of ERV1tg mice may be skewed to patrolling (CCR2^-^CX3CR1^high^, M2-like) relative to inflammatory (CCR2^+^CX3CR1^int^, M1-like) phenotype under dietary fat overload^8,9^. We interrogated monocyte phenotype changes induced by HFD feeding for 16 weeks in ERV1tg and WT mice by acoustic focusing flow cytometry (Fig. 3a). In steady state, ERV1tg mice have higher numbers of circulating monocytes (Fig. 3c), higher M2-to-M1 monocyte ratio (Fig. 3e) and less activated granulocytes (GC) as measured by CD11b levels (Fig. 3f). The expression of ERV1 in peripheral blood leukocytes was significantly higher in monocytes compared to GC, and at similar levels in both patrolling and inflammatory monocytes (Figure S2A). HFD feeding induced an increase in GC numbers in both ERV1tg and WT mice (Fig. 3d) and in monocyte numbers In WT (Fig. 3c). A lower proportion of M1-like monocytes were CCR2^high^ and a higher proportion of M2-like monocytes were CX_3_CR1^high^ in ERV1tg mice (Fig. 3g,h). HFD feeding induced a 30% increase of CCR2 and 20% decrease of CX_3_CR1 in monocytes of WT mice. In ERV1tg mice however, the expression of CCR2 was reduced by 40% regardless of diet and that of CX_3_CR1 increased by 30% on monocytes of mice on HFD, compared to WT (Fig. 3i). Further, the M1-to-M2 ratio correlated strongly with body weight in ERV1tg mice (Fig. 3j). Similar to peripheral blood, peritoneal elicited macrophage expressed higher ERV1 levels compared to GC (Figure S2B). These findings suggested that skewing of monocytes/macrophages to M2-like in ERV1tg mice contribute to the reduced liver and VAT inflammation, and to reduced body weight gain while under dietary fat overload. To further corroborate this notion, we assessed VAT and liver macrophages for expression of iNOS (M1 activation marker) and CD206 (M2 activation marker) by immunofluorescence. Most macrophages (F4/80^+^) in VAT of WT mice on HFD were positive for iNOS, and fewer for CD206. One the other hand, most VAT macrophages of ERV1tg mice on HFD were positive for CD206, and fewer for iNOS (Fig. 3k). Analysis of VAT and liver by immunohistochemistry confirmed high ERV1 expression in ERV1tg mice on HFD (Fig. 4a,b). Further, expression of M2 activation markers YM1 and CD163 was also higher in ERV1tg mice (Fig. 4c–f). Notably, in the absence of metabolic overload ERV1 and YM1 expression in VAT of ERV1tg was higher compared to WT (Figure S3). Taken together, these findings indicate a protective role of the predominant patrolling/M2-like phenotype of monocytes/macrophages observed in ERV1tg mice in dietary fat overload conditions.

**Figure 5:**
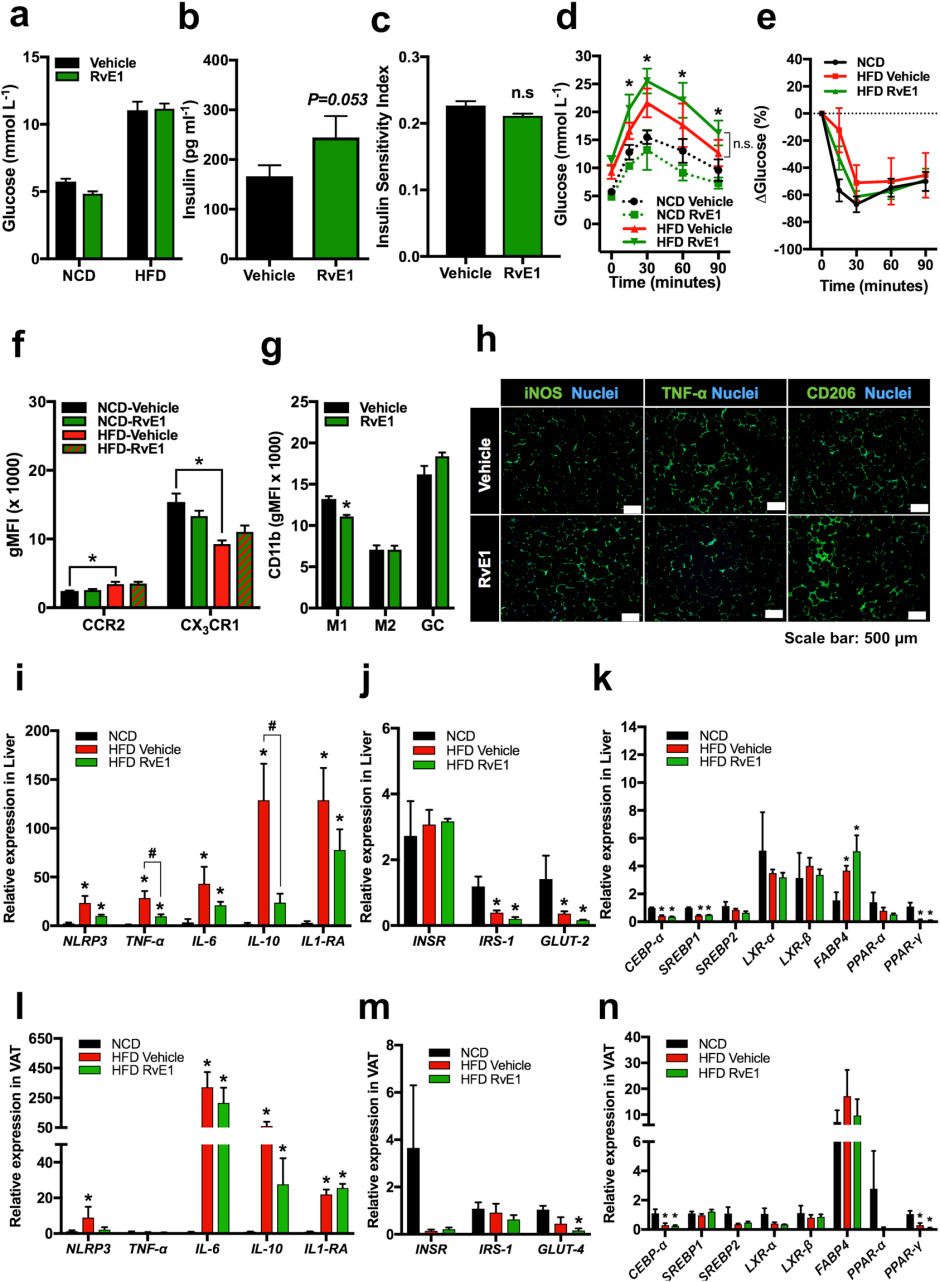
RvE1 treatment does not improve metabolic parameters in HFD induced obesity but stimulates M2 polarization and reduces upregulation of inflammatory genes in liver. WT male mice on HFD for 14 weeks were treated with Resolvin E1 (RvE1) i.v. (2 ng/g body weight) or vehicle (1% ethanol in normal saline) twice a week for 4 weeks. Fasting glucose (**a**) and insulin (**b**) were measured as described in Methods. (**c**) Insulin sensitivity index (QUICKI). GTT (**d**) and ITT (**e**) were performed at 18 weeks on HFD. (**b**) CCR2 and CX_3_CR1 expression was measured on peripheral blood monocytes and CD11b (**g**) on myeloid cells of HFD fed mice treated with RvE1 (n = 4 mice per group, *P < 0.05, unpaired *t* tests, Holm-Sidak). (**h**) Representative immunofluorescence micrographs of M1 (iNOS, TNF-α) and M2 (CD206) markers in VAT of vehicle and RvE1 treated mice on HFD. (**i**–**n**) Expression of inflammatory, glucose and lipid metabolism genes in liver (**i–k**) and VAT (**l–n**) of HFD fed mice on RvE1 treatment compared to NCD fed mice (n = 4 mice per group, *P < 0.05 vs NCD, ^#^*P* < 0.05 RvE1 vs Vehicle unpaired *t* tests). Histograms represent mean ± SEM.

We next investigated whether RvE1, a natural agonist of ERV1, can reverse the phenotype of WT mice on HFD for 14 weeks, after onset of chronic hyperglycemia and impaired insulin sensitivity. Mice were treated with RvE1 (2 ng/g body weight) or vehicle (1% ethanol in normal saline) twice weekly for 4 weeks. RvE1 partially reversed diet induced downregulation of CX_3_CR1 on monocytes (20% increase), downregulated CD11b on M1 (16% decrease) and was associated with predominant M2 polarization of ATM (Fig. 5f–h) although it did not significantly improve glucose tolerance or insulin sensitivity (Fig. 5a–e). Further, mRNA levels for *TNF-α* and *IL-10* were significantly reduced in liver and *NLRP3* was reduced in VAT of RvE1 treated mice (Fig. 5i), without significant differences in expression of insulin and glucose response genes, or lipid metabolism genes compared to vehicle treated mice (Fig. 5j–n). Fasting insulin levels, islet area and insulin to glucagon positive cell ratio also increased with RvE1 treatment (Figs 5b and S4b,d) but no differences were noted in hepatic steatosis or VAT morphology (Figure S4e–h). Together, these data indicate that, after onset of diet induced glucose intolerance and insulin resistance, RvE1 treatment may control inflammation in primary organs regulating glucose and lipid metabolism.

### ERV1tg mice produce more SPMs in steady state and during dietary overload

**Figure 6:**
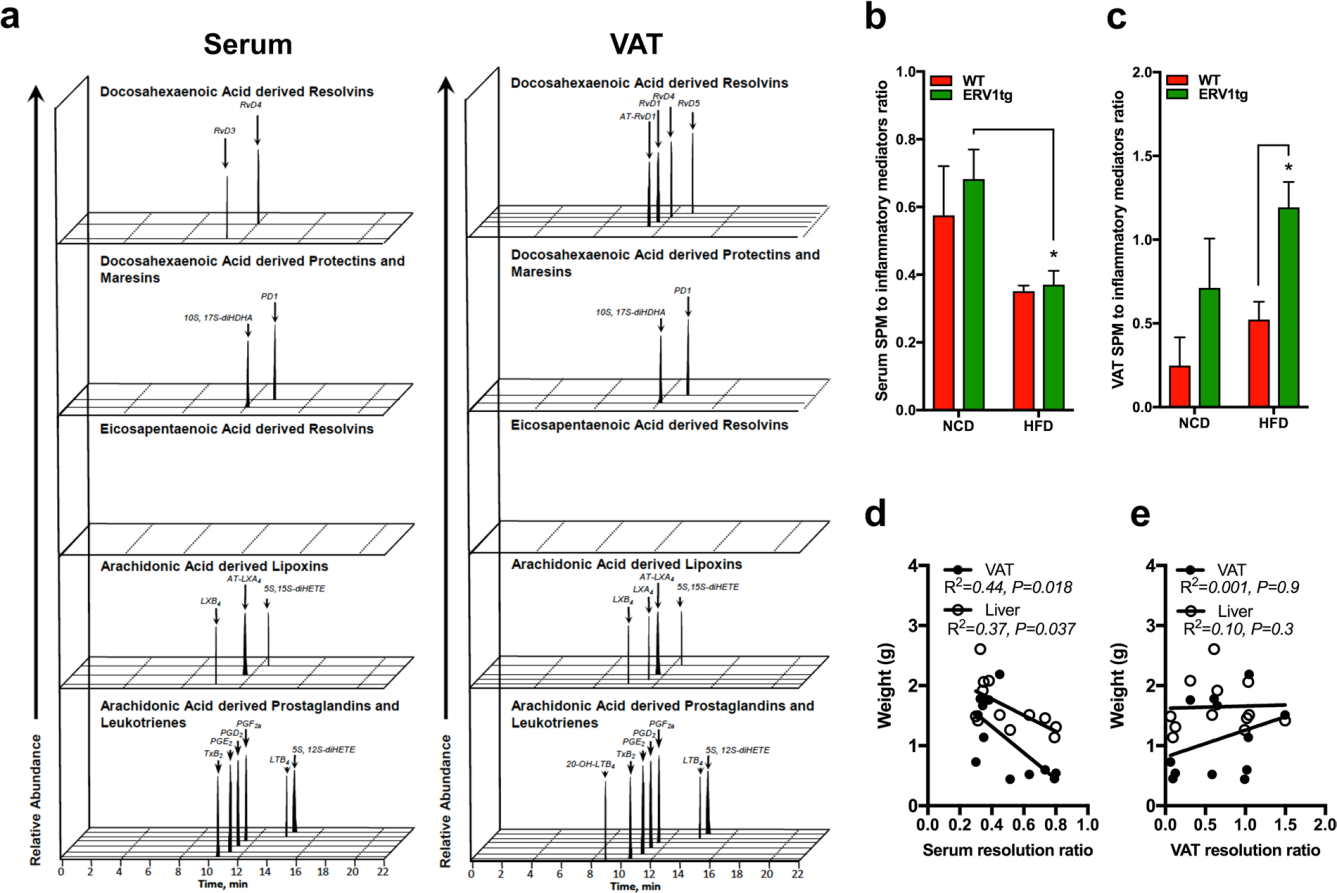
Myeloid ERV1 overexpression is associated with enhanced production of specialized pro-resoling lipid mediators (SPMs) in serum and VAT. Fasting serum and VAT from male mice on HFD or NCD for 20 weeks were analyzed by LC-MS/MS metabololipidomics for identification and quantification of lipid mediators of inflammation. (**a**) Representative multiple reaction monitoring (MRM) chromatograms for each of the identified lipid mediators in serum and VAT. Serum (**b**) and VAT (**c**) resolution ratios calculated by dividing the quantity of SPMs (lipoxins, resolvins, protectins) by the quantity of pro-inflammatory lipid mediators (LTB4, PGE2) (n = 3 mice per group, *P < 0.05 HFD vs. NCD for serum and ERV1tg vs. WT for VAT, unpaired *t* tests). (**d**,**e**) Correlations between serum (**d**) and VAT (**e**) resolution ratios and weights of liver and VAT in WT and ERV1tg mice on NCD and HFD (Pearson, n = 6 mice per genotype). Histograms represent mean ± SEM.

Collectively, monocyte skewing to M2-like and reduced inflammation in VAT and liver is associated with RvE1-ERV1 activation. Prevention of deregulated glucose metabolism under dietary overload in ERV1tg mice fed HFD suggests that ERV1 may trigger inflammation resolution programming orchestrated by monocytes/macrophages in obesity. We therefore quantified the SPM production by ERV1tg mice in steady state or on HFD by metabololipidomics targeting EPA, DHA and arachidonic acid (AA) derivatives. Both the NCD and HFD contain approximately 1% ω-3 PUFAs, including ALA, EPA and DHA, and <0.01% AA. Serum and VAT of mice on HFD for 20 weeks were subjected to liquid chromatography-tandem mass spectrometry (LC–MS/MS) for targeted lipid mediator analysis. SPMs were identified in both serum and VAT from the DHA and AA metabolomes (Fig. 6a). These SPMs, namely lipoxin A4 (LXA_4_), aspirin triggered LXA_4_ (AT-LXA_4_), RvD4, RvD5, 5 S, 15S-diHETE, 10 S,17S-diHDHA and PD1 were higher in ERV1tg mice in steady state compared to WT (Table S2). The increased SPM levels were associated with higher leukotriene B4 (LTB_4_) and prostaglandin E2 (PGE2) levels in both serum and VAT of ERV1tg mice compared to WT (Figure S5). HFD feeding induced a general decrease in SPM serum levels, as well as VAT LXB_4_, RvD1, AT-RvD1, and RvD4 but also an increase in VAT 5 S,15S-diHETE and 10 S,17S-diHDHA. In comparison to WT, HFD feeding of ERV1tg mice was associated with an increase in VAT AT-LXA_4_, RvD5 and PD1 (Figure S5b,d). Since samples collected from multiple mice per condition were analyzed individually we were able to assess per subject the overall balance between pro-inflammatory lipid mediators and SPMs, that we called resolution ratio, and its relationship to the fat pad and liver weights. We calculated the resolution ratio by dividing the sum of SPMs (lipoxins, resolvins and protectins detected) by the sum of PGE2 and LTB_4_, in serum and in VAT. The serum resolution ratio was decreased on HFD compared to NCD for both genotypes (Fig. 6b). The VAT resolution ratio was increased on HFD in ERV1tg compared to WT (Fig. 6b,c). To further interrogate these findings, we assessed the correlations between serum and VAT resolution ratios and weights of liver and VAT fat pads. Significant inverse relationships between serum but not VAT resolution ratios and weight of both liver and VAT were noted (Fig. 6d,e). These findings suggest that ERV1 overexpression in myeloid cells does activate resolution programming, which in many respects is maintained under dietary overload conditions. They further indicate that systemic activation of resolution may be beneficial to prevent ectopic lipid accumulation, such as hepatic steatosis, in obese prone circumstances.

## Discussion

CCR2^+^ monocytes and adipose tissue macrophages activated to a pro-inflammatory M1-like phenotype play critical roles in onset and progression of type 2 diabetes and associated complications by sustaining non-resolving inflammation and insulin resistance^6,8,10,48,49^. ERV1 activation has shown potential to induce inflammation resolution through activation of monocytes/macrophages to M2-like phenotype, and its deletion was associated with glucose intolerance^34,50,51^. We investigated the impact of myeloid ERV1 activation on inflammation and metabolic imbalance in obesity. Our data shows that overexpression of ERV1 in myeloid cells prevents morbid body weight gain, glucose intolerance and hyperglycemia induced by high fat diet. The increased proportion of patrolling CX_3_CR1^high^ monocytes and reduction of CCR2 on inflammatory monocytes in ERV1tg mice was associated with protective changes in inflammatory, glucose and insulin response, and lipid metabolism genes in liver and adipose tissue. HFD feeding induced CCR2 upregulation and CX_3_CR1 downregulation in monocytes of wild type but not ERV1tg mice, and RvE1 treatment partially reversed these HFD-induced changes in monocytes. Further, both ERV1 overexpression and RvE1 treatment had a beneficial impact on diet induced changes in pancreatic islets and fasting serum insulin levels. Finally, ERV1tg mice produced more SPMs in serum and VAT, and maintained a higher SPM-to-inflammatory lipid mediator (resolution) ratio on HFD compared to wild type mice. The serum resolution ratio correlated with both liver and VAT size regardless of genotype or diet. Taken together these studies reveal an important role of systemic activation of inflammation resolution programs governed by myeloid cells in regulating metabolic imbalance in obese prone conditions.

Calorie imbalance generated by dietary fat overload leads to ectopic lipid accumulation in liver, and adipocyte dysfunction that induces macrophage infiltration and favors lipolysis in white adipose tissue^52^. The increased fatty acid flux to liver promotes esterification and triglyceride synthesis further exacerbating hepatic steatosis. These hepatic processes are substrate driven and independent of liver but dependent on VAT insulin signaling. In this context, the ability of insulin to acutely regulate hepatic gluconeogenesis and prevent hyperglycemia occurs mostly by an indirect mechanism through inhibition of white adipose tissue lipolysis^53,54^. Polarization of macrophages to M1 phenotype contributes to imbalance between VAT lipogenesis and lipolysis in favor of the latter, as well as to hepatic steatosis, both being driven by inflammation mediated insulin resistance^11,55,56^. Our findings of reduced liver and VAT inflammation combined with unaltered lipogenesis and reduced hepatic steatosis in ERV1tg mice on HFD indicate that the induction of monocyte/macrophage shifts to M2-like phenotypes results in physiological processing of excess fat. This is likely mediated by extending the life span and function of patrolling monocytes via CX_3_CR1. Two single-nucleotide polymorphisms (T280M and V249I), located in the coding sequence of human *CX3CR1*, which impact CX_3_CR1 expression and function, were associated with increased incidence of type 2 diabetes and metabolic syndrome, partially explained by altered monocyte-adipocyte interactions^57,58^. The higher proportion of CX_3_CR1^high^ patrolling monocytes in ERV1tg mice may be responsible for the overall higher number of monocytes since these cells have longer lifespan compared to inflammatory monocytes. Indeed, recent evidence showed that CX_3_CR1 plays a critical role in extending the life span of monocytes and that inflammatory CCR2^+^CX_3_CR1^int^ monocytes are obligate precursors for patrolling CCR2^-^CX_3_CR1^high^ monocytes in the bone marrow and blood under homeostasis^12,13^. Therefore, enhancing surveillance of metabolic overload by CCR2^-^CX_3_CR1^high^ monocytes seems a potent therapeutic approach to prevent obesity associated morbidities. The regulatory impact of ERV1 on CCR2 and CX_3_CR1 in monocytes favored patrolling over inflammatory phenotype in both steady state and during dietary fat overload conditions, with a net positive metabolic impact reflected by prevention of hyperglycemia.

The predominant activation of adipose tissue macrophage to M2-like phenotype observed in ERV1tg mice on HFD was associated with peri-adipocyte fibrosis. Whether fibrosis in adipose tissue was protective or pathogenic in ERV1tg mice is unclear. Peri-adipocyte fibrosis is often associated with unfavorable phenotypes, but it may also represent a failed resolution attempt by the body when activation of adipose tissue macrophages to M2-like phenotype is not associated with reduced inflammation. In our study, the observed peri-adipocyte fibrosis was associated with an increase in expression of the anti-inflammatory cytokine IL-1RA and downregulation of inflammatory genes compared to WT. We found these findings indicative of successful control of adipose tissue inflammation and healthy-type hypertrophy/hyperplasia of adipocytes. A recent study on visceral and subcutaneous adipose tissues from humans with obesity has found that fibrosis was decreased, hypertrophy increased and preadipocyte frequency decreased in patients with diabetes when compared with non-diabetic subjects, supporting the hypothesis that adipose tissue fibrosis in the context of obesity limits pathologic expansion with beneficial outcomes on systemic metabolism, thus preventing the onset of diabetes^59^.

While adequate processing of excess lipids and carbohydrates is central to maintaining normal glucose levels and insulin sensitivity in fasted and fed states, appropriate insulin secretion and action is necessary to achieve metabolic balance. The fractalkine/CX_3_CR1 system was shown to play an important role in regulation of insulin secretion^60,61^. However, these studies have not addressed the contribution of monocyte CX_3_CR1 in regulating β-cell function but confirmed the impaired insulin secretion in CX_3_CR1 knockout mice without altered intra-islet vascularization. This suggests that both monocyte/macrophage and β-cell CX_3_CR1 may be involved in regulation of insulin secretion. On the one hand, the increase in fasting serum insulin levels and maintenance of high insulin to glucagon ratio in pancreatic islets by HFD fed ERV1tg mice in the absence of hyperglycemia suggest a potent β-cell response to episodes of transient hyperglycemia combined with some degree of peripheral insulin resistance. The increased proportion of CX_3_CR1^high^ monocytes in ERV1tg may in part explain these findings. Further investigations into the roles of this monocyte population in pancreatic inflammation and insulin secretion are needed to elucidate its implications in β-cell function. On the other hand, the delayed response to administered insulin in ERV1tg mice on either diet may be explained by the activation of a cathecolamine-mediated counter-regulatory response to insulin or different insulin absorption rate in ERV1tg mice compared to WT mice. Catecholamine production by alternatively activated macrophages has been implicated in adaptive thermogenesis, and therefore ERV1tg mice may exhibit a robust macrophage catecholamine response to stress, such as restraining for insulin administration^62^. In insulin-induced hypoglycemic clamp experiments, FVB mice were shown to mount a potent endocrine counter-regulatory response through increased secretion of catecholamines and glucagon, compared to other strains, suggesting a tight control of insulin secretion in this strain^63^. A future study should address the role of myeloid ERV1 in adaptive thermogenesis, as well as its impact on the response to exogenous insulin and the endogenous insulin secretory response through euglycemic-hyperinsulinemic clamp and hyperglycemic clamp experiments.

The metabolic imbalance associated with obesity is an inflammatory trigger that can be reversed by body weight loss. In the absence of excess calorie use or storage this metabolic insult is sustained by adipocytes and hepatocytes whose response begins the inflammatory program, mediating the interface between metabolic input and inflammatory output, and leading to a chronic unresolved inflammatory state^64^. While the mechanisms of failed resolution associated with obesity and insulin resistance are incompletely understood, mounting evidence suggests that excess saturated free fatty acids, increased gut permeability and dysbiosis, persistence of hyperglycemia, hypoxia and tissue damage contribute to resolution failure^49,65^. On the one hand, free fatty acids drive inflammatory signaling through NLRP3 inflammasome activation and upregulation of cyclooxygenase 2 (COX-2) with subsequent production of pro-inflammatory cytokines and prostaglandins in macrophages, which impair phagocytosis in an autocrine manner^66,67^. On the other hand, ω-3 PUFAs and derived SPMs enhance macrophage clearing function in the context of obesity^26,31,33^. Since SPMs are biosynthesized during macrophage phagocytosis, it is possible that substrate diversion to proresolving lipid mediators may partly underlie the protective effects of ω-3 PUFAs. Our finding of increased SPM production in ERV1tg mice corroborated with significant correlations between serum SPM vs. inflammatory lipid mediator ratio and the size of metabolic organs, liver and VAT, support this hypothesis. The upregulation of *SREBP2* and *PPAR-α* in liver and downregulation of *FABP4* in VAT of ERV1tg indicate enhanced fatty acid oxidation and cholesterol removal from liver, and storage of fatty acids in adipocytes. Further, *SREBP1*, *LXR* and *PPAR-α* were shown to induce expression of desaturases and elongases involved in endogenous synthesis of long chain PUFAs including EPA and DHA^68^. This indicates increased catabolism of excess saturated free fatty acids and enhanced synthesis of ω-3 PUFAs in ERV1tg mice. Therefore, a switch to ω-3 PUFAs synthesis and metabolism could explain the higher levels of SPMs in these mice. Altogether these findings indicate that ERV1 overexpression in myeloid cells activates resolution programs that deal with dietary overload through promotion of excess nutrient storage or removal, enhanced metabolism of ω-3s PUFAs and prevention of inflammation in central metabolic organs.

In wild type mice on HFD for 14 weeks RvE1 treatment had the same impact on monocytes and inflammation as ERV1 overexpression suggesting that direct ligand binding is needed to induce phenotypic shifts in circulating monocytes. RvE1 binds to ERV1 on monocytes and macrophages and induces inflammation resolution signal transduction by attenuating NF-κB activation and enhancing phagocytosis^69–71^. The pro-phagocytic actions of RvE1 on myeloid cells are likely mediated through ribosomal protein S6 (rS6) and mechanistic target of rapamycin (mTOR)^69,72^. As opposed to ERV1tg mice, metabolic parameters were not significantly improved with RvE1 treatment in WT after onset of obesity and insulin resistance. Higher RvE1 doses and/or daily delivery may be needed to improve metabolic parameters in obesity induced insulin resistance^31^.

The results herein demonstrate that activation of resolution programs in myeloid cells is beneficial in dietary fat overload conditions. These findings further our understanding of peripheral blood monocyte phenotypes associated with diet-induced obesity and means to modulate metabolic imbalance and inflammation resolution. Emerging evidence points to the importance of addressing chronic un-resolved low grade inflammation associated with insulin resistance and type 2 diabetes. Although therapeutic approaches to inflammation in diabetes are separated from those aiming at controlling blood glucose levels, this study shows that prevention of hyperglycemia can be achieved by enhancing the pro-resolution patrolling monocytes in obese prone environments.

## Electronic supplementary material

Supplemental information
