# Author Correction: ERV1 Overexpression in Myeloid Cells Protects against High Fat Diet Induced Obesity and Glucose Intolerance

**DOI:** 10.1038/s41598-018-22520-5

**Published:** 2018-03-02

**Authors:** Corneliu Sima, Eduardo Montero, Daniel Nguyen, Marcelo Freire, Paul Norris, Charles N. Serhan, Thomas E. Van Dyke

**Affiliations:** 1 Center for Clinical and Translational Research, The Forsyth Institute, 245 First Street, Cambridge, MA 02138 USA; 2 Department of Oral Medicine, Infection and Immunity, Harvard School of Dental Medicine, 188 Longwood Ave, Boston, MA 02115 USA; 3 Section of Graduate Periodontology, Faculty of Odontology, University Complutense of Madrid, Pza. Ramón y Cajal s/n, Madrid, 28040 Spain; 4 Center for Experimental Therapeutics and Reperfusion Injury, Department of Anesthesiology, Perioperative and Pain Medicine, Brigham and Women’s Hospital, and Harvard Medical School, 60 Fenwood Road, Boston, MA 02115 USA

Correction to: *Scientific Reports* 10.1038/s41598-017-13185-7, published online 09 October 2017

In this Article, Figure 1e is a duplication of Figure 3j. The correct Figure 1 appears below as Figure 1.

**Figure 1:**
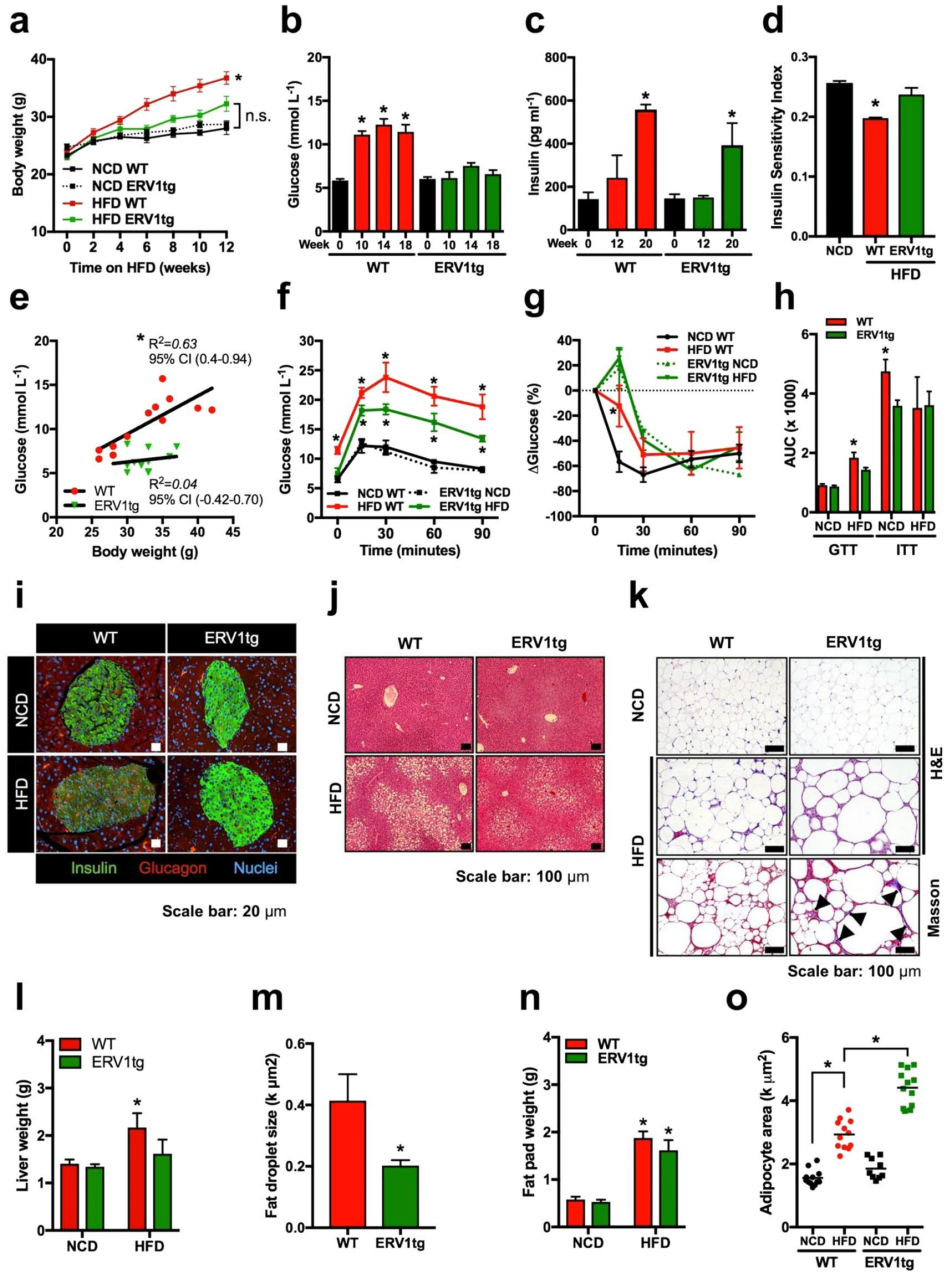
ERV1 transgenic mice are protected from diet induced obesity, hepatic steatosis and glucose intolerance. (**a**) Body weights of ≥6 weeks-old male FVB mice on normal control diet (NCD) or high fat diet (HFD) for 12 weeks (n = 12 NCD per group; *p = 0.0075, ANOVA repeated measures test; F (1.126, 6.755) = 13.56). (**b**,**c**) Fasting blood glucose and serum insulin were measured in WT (n = 12) and ERV1tg (n = 8) mice for 20 weeks on HFD (*p < 0.05, unpaired t-tests vs. baseline). (**d**) Quantitative insulin sensitivity check index (QUICKI) for mice on NCD or HFD for 16 weeks (n = 8 mice/group, *P < 0.05, t test). (**e**) Correlation between fasting blood glucose and body weight in mice on NCD or HFD for 16–20 weeks (*Pearson, P < 0.01, n = 12 mice/group). (**f**,**g**) Glucose (**f**) and insulin (**g**) tolerance in NCD- or HFD-fed WT and ERV1tg mice (n = 4 per group, *P < 0.05 HFD vs. NCD, t tests, Holm-Sidak correction for multiple comparisons assuming unequal variance per time point (P < 0.01), one of two similar experiments). (**h**) AUC for glucose (GTT) and insulin (ITT) tolerance test results for WT and ERV1tg mice (n = 4 per group, *P < 0.05 HFD vs NCD, t tests). (**i**) Representative glucagon and insulin immunofluorescence micrographs of pancreatic islets from WT and ERV1tg mice on NCD or HFD for 18 weeks. (**j**,**k**) Representative micrographs of liver (**j**) and VAT (**k**) stained with hematoxylin and eosin, and Masson’s trichrome (**k**, Bottom row; arrows indicate collagen). (**l**,**n**) Weights of livers (**l**) and single epididymal VAT (**n**) of WT and ERV1tg mice on NCD or HFD for 18 weeks (n = 4 mice/group, *P < 0.05 HFD vs. NCD, t test). (**m**) Fat droplet size in liver of WT and ERV1tg mice on HFD for 18 weeks (n = 4 mice per group, *P < 0.05, t test). (**o**) Adipocyte size in VAT of mice on HFD or NCD (n = 4 per mice group, *P < 0.05 t tests). All t tests were unpaired and two tailed. Histograms represent mean ± SEM.

