# Supplementary material for: ERV1 Overexpression in Myeloid Cells Protects against High Fat Diet Induced Obesity and Glucose Intolerance
Source: Sci Rep. 2017 Oct 9;7:12848. doi: 10.1038/s41598-017-13185-7 (PMC5634420; doi:10.1038/s41598-017-13185-7)
Supplement: Supplementary file 1 — Supplemental information [file 41598_2017_13185_MOESM1_ESM.pdf]

Supplementary Information

ERV1 Overexpression in Myeloid Cells Protects against High Fat Diet Induced Obesity and Glucose Intolerance

Corneliu Sima[1,2], Eduardo Montero[3], Daniel Nguyen[1,], Marcelo Freire[1,2], Paul Norris[4], Chales N. Serhan[4,5] and Thomas E. Van Dyke[1,2]

[1] Center for Clinical and Translation Research, The Forsyth Institute, 245 First Street, Cambridge, MA, 02138, USA

[2] Department of Oral Medicine, Infection and Immunity, Harvard School of Dental Medicine, 188 Longwood Ave, Boston, MA, 02115, USA

[3] Section of Graduate Periodontology, Faculty of Odontology, University Complutense of Madrid, Pza. Ramón y Cajal s/n, Madrid, 28040, Spain

[4] Center for Experimental Therapeutics and Reperfusion Injury Brigham and Women's Hospital, 60 Fenwood Rd, Boston, MA 02115, USA

[5] Department of Anesthesiology, Perioperative and Pain Medicine, Harvard Institutes of Medicine, Harvard Medical School, 25 Shattuck St, Boston, MA 02115

Number of figures: 5

Number of tables: 2

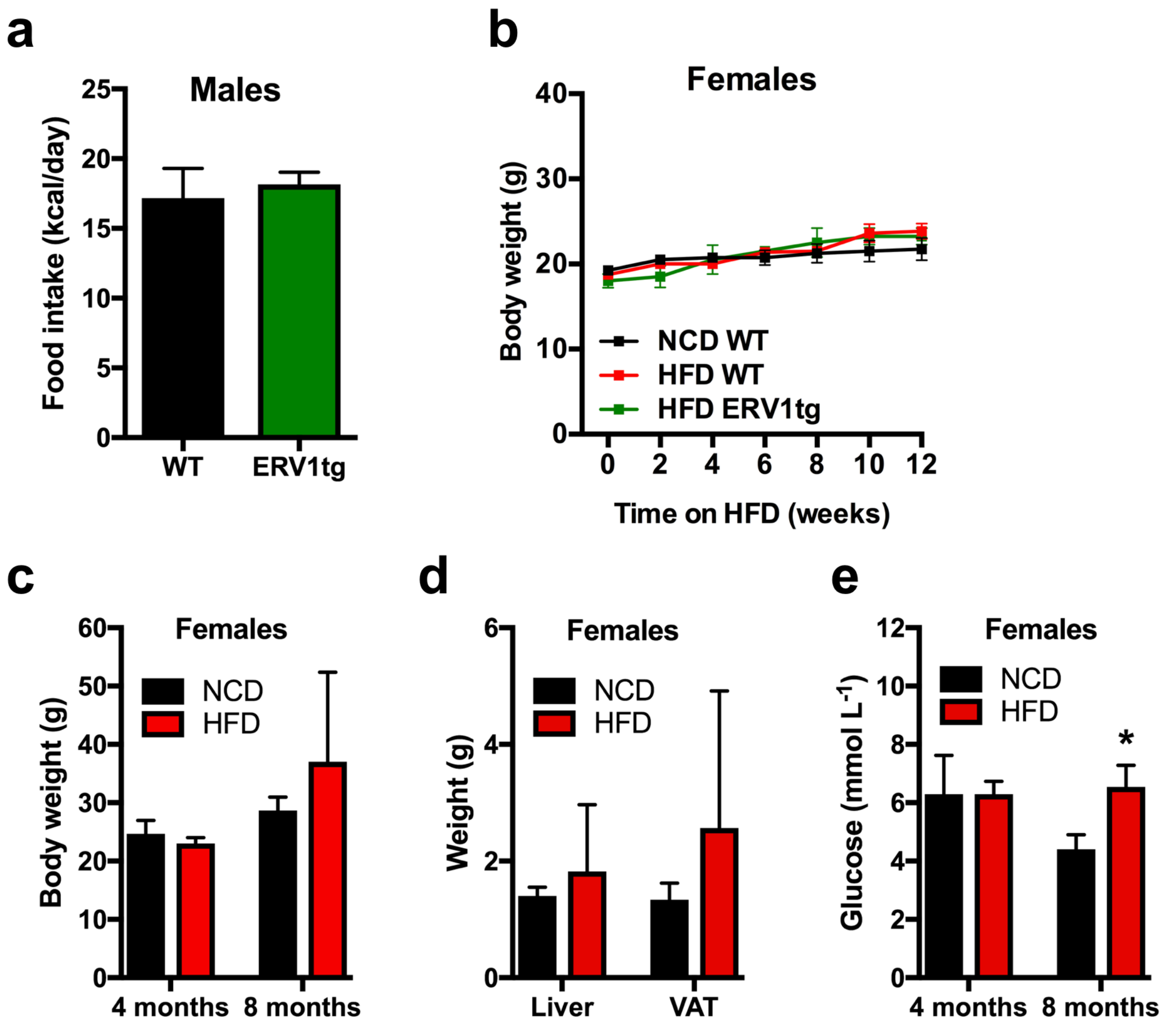

Figure S1. ERV1tg and WT females do not gain significant weight after 8 months on HFD. (**a**) Food intake was measured in WT and ERV1tg male mice between weeks 2-12 on HFD (n=8 mice per group). (**b**) Female WT and ERV1tg mice on HFD were monitored for changes in body weight every 2 weeks for 3 months, and subsequently at 4 and 8 months (n=4 mice per group) (**c**). Liver and VAT were measured at 8 months (**d**) and glucose at 4 and 8 months on the diet (**e**). Histograms represent mean ± SEM.

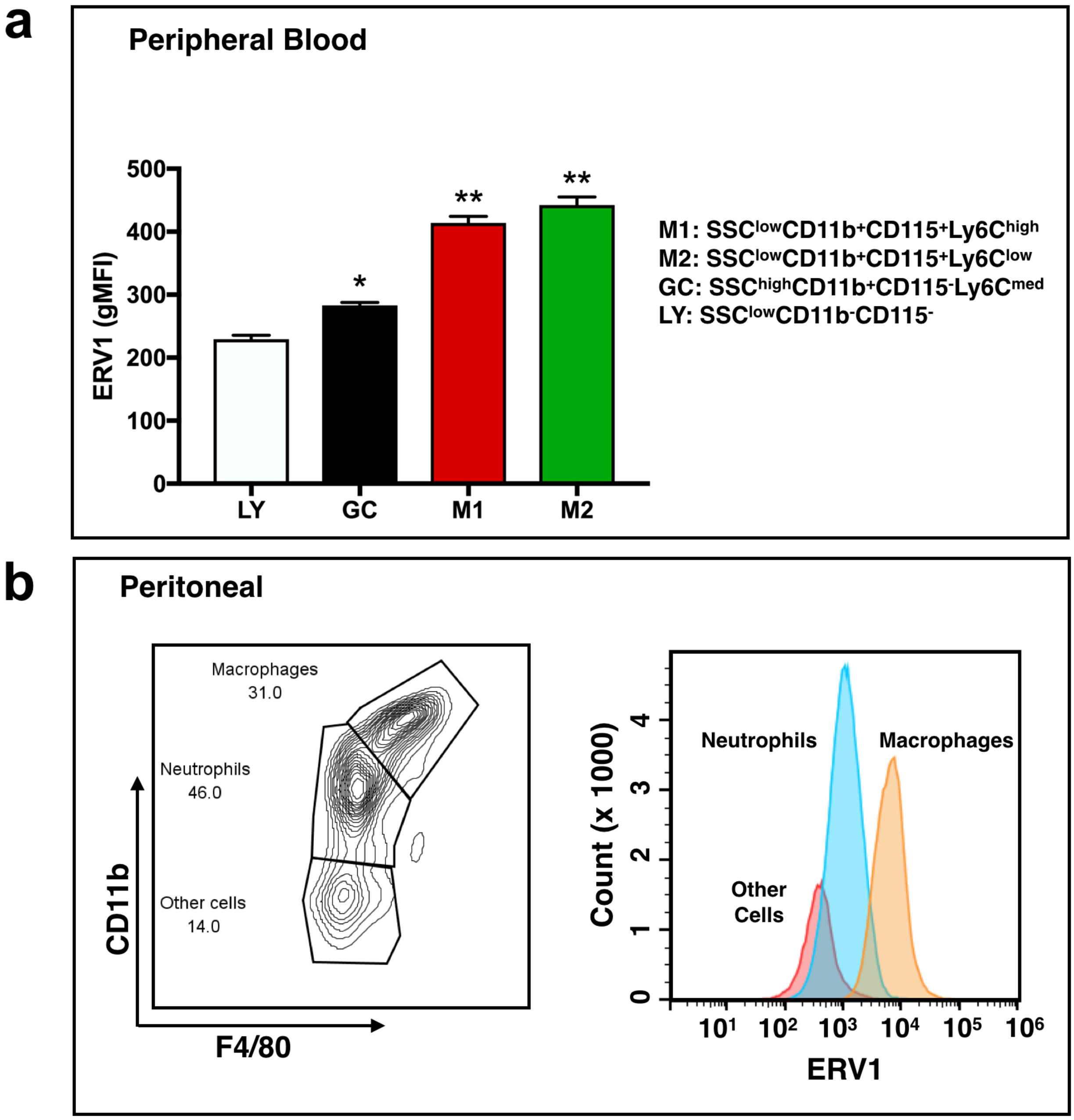

Figure S2

Figure S2. ERV1 expression is increased in peripheral blood monocytes and tissue macrophages compared to granulocytes (**a**): Peripheral blood immunophenotyping for ERV1 revealed increased expression on monocytes compared to granulocytes, and similar expression on $CD115^{+}Ly6C^{high}$ (M1) and $CD115^{+}Ly6C^{low}$ (M2) monocytes in ERV1tg mice (*p<0.05, GC vs. LY, *t*-test; **p<0.05 vs. GC, *t*-test). (**b**): The expression of ERV1 on peritoneal zymosan A elicited leukocytes (18 h post-induction) revealed higher expression on macrophages compared to neutrophils, and myeloid cells ($CD11b^{+}$) more than other cells (representative of 3 ERV1tg mice). GC, granulocytes; LY, lymphocytic cells.

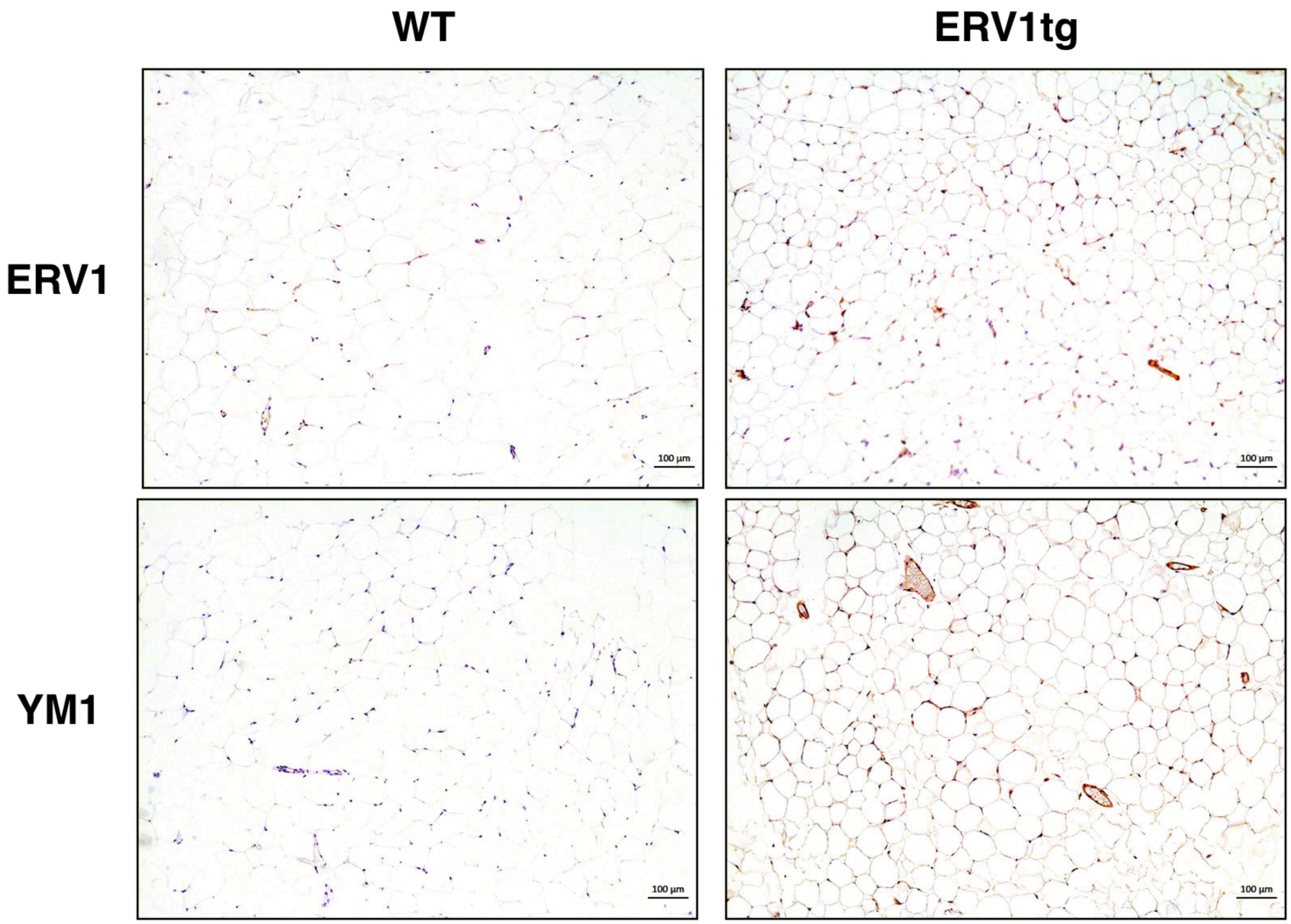

Figure S3

Figure S3. **ERV1 and YM1 expression in VAT of male WT and ERV1tg mice.** VAT samples from aged matched male WT and ERV1tg mice on NCD were assessed by immunohistochemistry for ERV1 and YM1 expression. Following de-paraffinization, antigen retrieval (baking), and blocking (serum) sections were stained with rabbit anti-hERV1 (1:2000) and anti-mYM1 (1:500) primary antibodies overnight followed by biotinylated goat anti-rabbit secondary antibody incubation.

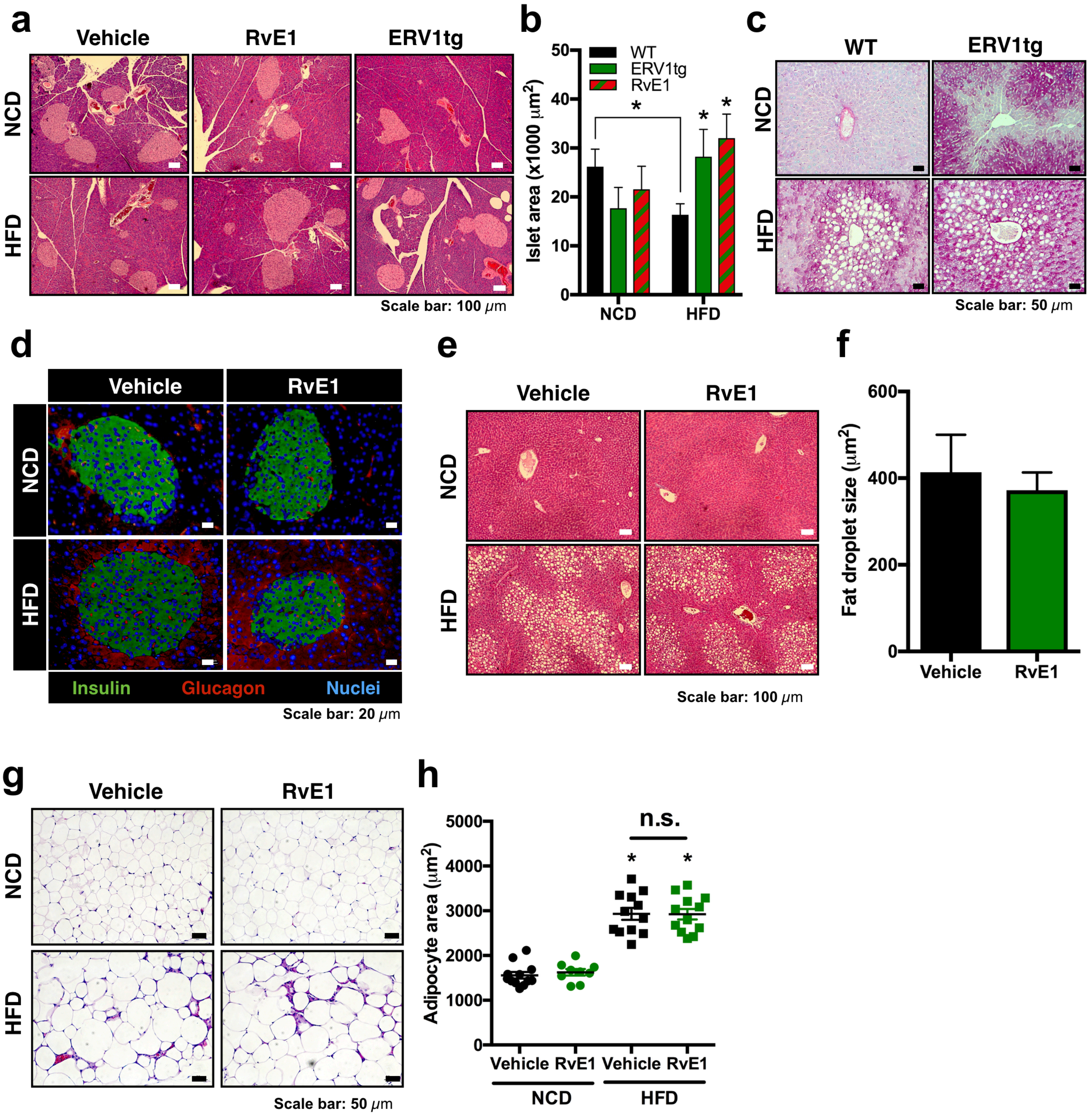
a
Vehicle
RvE1
ERV1tg
NCD
HFD
Scale bar: 100 μm
b
WT
ERV1tg
RvE1
Islet area (x1000 μm2)
NCD
HFD
c
WT
ERV1tg
NCD
HFD
Scale bar: 50 μm
d
Vehicle
RvE1
NCD
HFD
Insulin
Glucagon
Nuclei
Scale bar: 20 μm
e
Vehicle
RvE1
NCD
HFD
Scale bar: 100 μm
f
Fat droplet size (μm2)
Vehicle
RvE1
g
Vehicle
RvE1
NCD
HFD
Scale bar: 50 μm
h
n.s.
Adipocyte area (μm2)
Vehicle RvE1 Vehicle RvE1
NCD
HFD

Figure S4

Figure S4. Pancreatic islet area, hepatic and VAT lipid accumulation in RvE1 treated mice. Male WT mice on HFD or NCD for 14 weeks were treated with RvE1 (2ng/g body weight) twice weekly for 4 weeks. (**a**) Representative micrographs of pancreas stained with hematoxylin and eosin (scale bar, 100 μm) (**b**) Pancreatic islet area was measured in 3 low power fields per sample (> 5 islets per sample) using Fiji (ImageJ, NIH) (One way ANOVA and unpaired *t* tests *P<0.05, n=4 mice per group). (**c**) Representative micrographs of ERV1tg and WT livers stained with PAS for glycogen. Diastase was used to confirm glycogen staining. (**d**) Representative glucagon and insulin immunofluorescence micrographs of pancreatic islets from WT treated with RvE1 or vehicle for 4 weeks. (**e**) Representative micrographs of liver stained with hematoxylin and eosin. (**f**) Fat droplet size in liver of WT on RvE1 or vehicle treatment (n=4 mice per group). (**g**) Representative micrographs of VAT stained with hematoxylin and eosin. (**h**) Adipocyte size in VAT of mice on RvE1 treatment (n=4 per mice group).

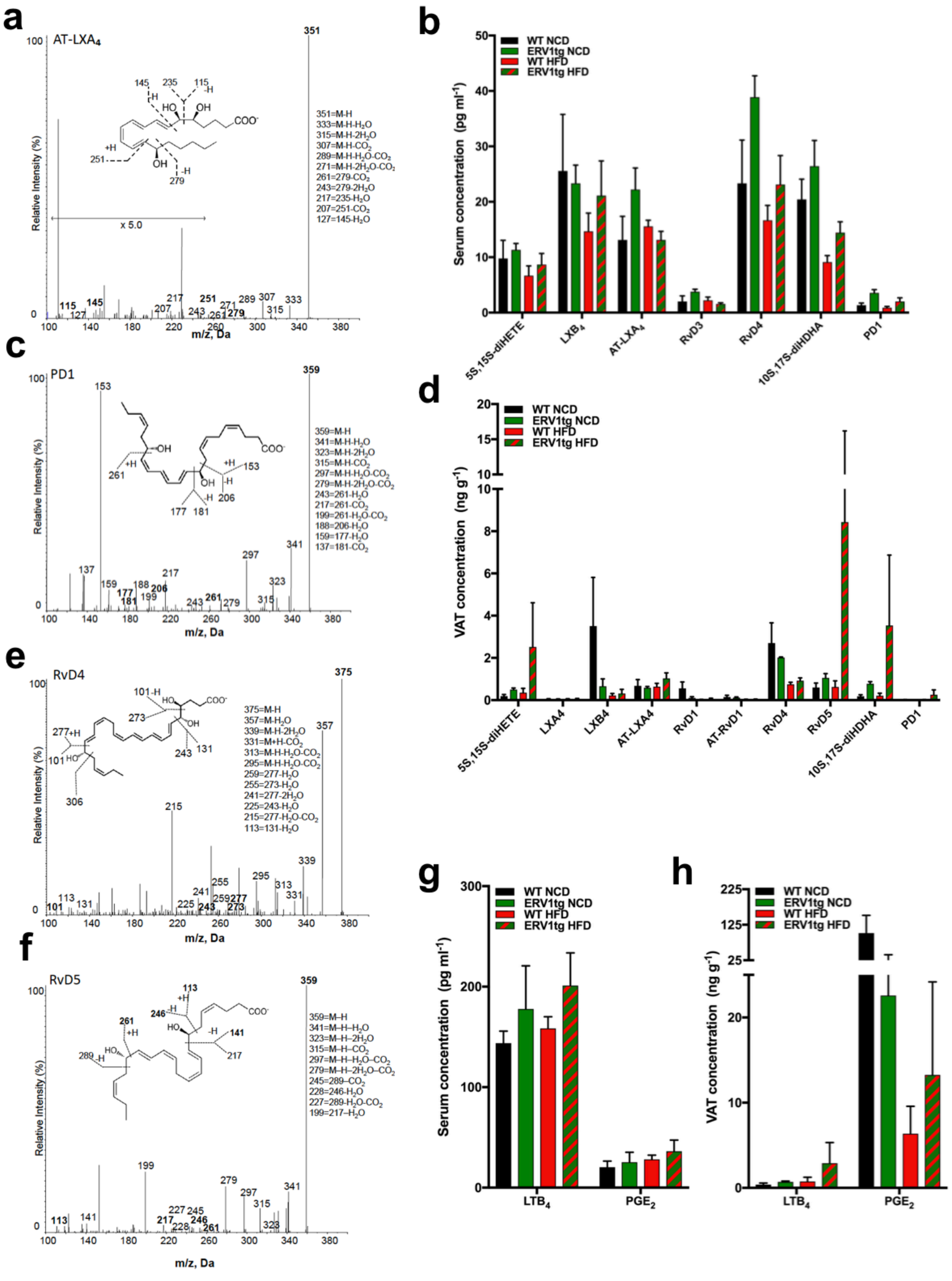
a
AT-LXA4
b
WT NCD
ERV1tg NCD
WT HFD
ERV1tg HFD
Serum concentration (pg ml-1)
c
PD1
d
VAT concentration (ng g-1)
e
RvD4
f
RvD5
g
h
LTB4
PGE2
Relative Intensity (%)
m/z, Da

Figure S5

Figure S5. Identification of enhanced production of specialized pro-resoling lipid mediators (SPMs) in serum and VAT of ERV1tg mice. MS-MS fragmentation spectra employed for identification of AT-LXA_4 (**a**) and PD1 (**c**) in serum and RvD4 (**e**) and RvD5 (**f**) in VAT. Serum SPM (**b**) and pro-inflammatory mediator (**g**) levels. VAT SPM (**d**) and pro-inflammatory mediator (**h**) levels (n=3 mice/group).

Duplex #	FAM	Assay ID	VIC	Assay ID
1	Pparg	Mm00440940_m1	GAPDH	4352339E
2	Nlrp3	Mm00840904_m1	Hprt	Mm03024075_m1
3	Insr	Mm01211875_m1	FABP4	Mm00445878_m1
4	Il10	Mm01288386_m1	Rn18s	Mm04277571_s1
5	Slc2a4	Mm00436615_m1	ACTB	Mm02619580_g1
6	Irs1	Mm01278327_m1	CEBPa	Mm00514283_s1
7	Tnf	Mm00443258_m1	SREBPF1	Mm00550338_m1
8	Slc2a2	Mm00446229_m1	NR1H2	Mm00437265_g1
9	Il6	Mm00446190_m1	NR1H3	Mm00443451_m1
10	Il1rn	Mm00446186_m1	SREBPF2	Mm01306292_m1
11	Ppara	Mm00440939_m1		

Table S1. Primers for quantitative PCR. Pre-designed target and endogenous Taqman gene primer/probe assays were purchased from ThermoFisher Scientific. The assays above were ran in duplex and confirmed by the manufacturer that no inter-assay primer interactions would occur. Real-time qPCR reaction wells contained 10 µl of TaqMan fast advanced master mix (Applied Biosystems), 1 µl of each Taqman assay, and 100 ng of cDNA sample template in 8 µl nuclease-free water. The reaction was carried out in a StepOnePlus real-time PCR system (Applied Biosystems) set at 50°C for 2 min, 95°C for 20 sec, then 40 cycles of 95° for 1 sec, 60° for 20 sec.

**Mediator**	**Serum**				**VAT**			
	NCD		HFD		NCD		HFD	
	WT	**ERV1tg**	WT	**ERV1tg**	WT	**ERV1tg**	WT	**ERV1tg**
LTB_4	144	**178**	158	**201**	389	**700**	749	**2902**
PGE_2	20	**25**	28	**36**	101728	**22590**	6355	**13265**
5S, 15S-diHETE	9.8	**11**	6.7	**8.7**	190	**498**	355	**2517**
10S, 17S-diHDHA	20	**26**	9.1	**14**	179	**778**	210	**3536**
LXA_4	-	**-**	-	**-**	44	**52**	52	**56**
LXB_4	26	**23**	15	**21**	3506	**655**	220	**312**
AT-LXA_4	135	**225**	155	**145**	675	**585**	645	**1025**
RvD1	-	**-**	-	**-**	557	**104**	31	**56**
AT-RvD1	-	**-**	-	**-**	150	**117**	29	**40**
RvD3	2	**3.78**	2.22	**1.56**	-	**-**	-	**-**
RvD4	23	**39**	17	**23**	2700	**2015**	749	**923**
RvD5	-	**-**	-	**-**	600	**1049**	622	**8432**
PD1	1.3	**3.6**	0.89	**2**	20	**30**	21	**251**

Table S2. Serum and VAT lipid mediators on NCD and HFD. Quantities are expressed as pg/mL for serum and pg/g tissue for VAT and represent means of n=3 samples per condition.
